# Analysis of Tumor Microenvironment Characteristics in Bladder Cancer: Implications for Immune Checkpoint Inhibitor Therapy

**DOI:** 10.3389/fimmu.2021.672158

**Published:** 2021-04-15

**Authors:** Xingyu Chen, Haotian Chen, Dong He, Yaxin Cheng, Yuxing Zhu, Mengqing Xiao, Hua Lan, Zhanwang Wang, Ke Cao

**Affiliations:** ^1^ The Third Xiangya Hospital, Central South University, Changsha, China; ^2^ The Second People’s Hospital of Hunan Province, Hunan University of Chinese Medicine, Changsha, China

**Keywords:** bladder cancer, tumor microenvironment, immune checkpoint, immunotherapy, tumor immune dysfunction and exclusion

## Abstract

The tumor microenvironment (TME) plays a crucial role in cancer progression and recent evidence has clarified its clinical significance in predicting outcomes and efficacy. However, there are no studies on the systematic analysis of TME characteristics in bladder cancer. In this study, we comprehensively evaluated the TME invasion pattern of bladder cancer in 1,889 patients, defined three different TME phenotypes, and found that different subtypes were associated with the clinical prognosis and pathological characteristics of bladder cancer. We further explored the signaling pathways, cancer-immunity cycle, copy number, and somatic mutation differences among the different subtypes and used the principal component analysis algorithm to calculate the immune cell (IC) score, a tool for comprehensive evaluation of TME. Univariate and multivariate Cox regression analyses showed that ICscore is a reliable and independent prognostic biomarker. In addition, the use of anti-programmed death-ligand (PD-L1) treatment cohort, receiver operating characteristic (ROC) curve, Tumor Immune Dysfunction and Exclusion (TIDE), Subnetwork Mappings in Alignment of Pathways (SubMAP), and other algorithms confirmed that ICscore is a reliable prognostic biomarker for immune checkpoint inhibitor response. Patients with higher ICscore showed a significant therapeutic advantage in immunotherapy. In conclusion, this study improves our understanding of the characteristics of TME infiltration in bladder cancer and provides guidance for more effective personalized immunotherapy strategies.

## Introduction

Bladder cancer is the ten most common cancer worldwide, is difficult to diagnose early, metastasizes rapidly, but currently has ineffective treatments (1, 2). Immune checkpoint therapy (ICT) is an immunotherapy that targets cytotoxic lymphocyte antigen-4 (CTLA-4), programmed cell death protein 1 (PD-1), or programmed death ligand 1 (PD-L1) (3–5). Immunotherapeutics for PD-1 or PD-L1 have greatly improved the survival of some patients and changed the intervention measures for advanced bladder cancer. However, most patients gain little to no clinical benefits from these immunotherapeutics (6, 7). Previous studies have found that PD-1 and PD-L1 expression, microsatellite instability status, and mutation load are not the best biomarkers for predicting immune checkpoint inhibitor responsiveness (8, 9). Therefore, it is necessary to establish new predictive indicators for checkpoint immunotherapy.

Tumor cells grow and survive in the tumor microenvironment (TME), which not only contains cancer cells, but also stromal cells such as resident fibroblasts (cancer-associated fibroblasts), macrophages, and recruited cells such as infiltrating immune cells (bone marrow cells and lymphocytes), bone marrow-derived cells (endothelial progenitor cells and hematopoietic progenitor cells), and secreted factors (cytokines, chemokines, and growth factors) (10, 11). These cancer cells trigger a variety of physiological changes through direct and indirect interactions with other TME components, such as inducing proliferation and angiogenesis, inhibiting apoptosis, avoiding hypoxia, and inducing immune tolerance. New evidence confirms that TME plays a key role in tumor progression, immune escape, and immunotherapeutic response (12–14). Therefore, a comprehensive analysis of the heterogeneity and complexity of TME is a key step to improve the success rate of existing ICTs and developing new immunotherapeutic strategies. A comprehensive analysis of the heterogeneity and complexity of TME will also be beneficial to determine the different tumor immunophenotypes and improve the ability to guide and predict the responsiveness of immunotherapy.

In this study, we aimed to identify novel biomarkers of bladder cancer using ICscore of various TME subtypes. The findings of this study might provide a better understanding of the molecular mechanisms of immune microenvironmental regulation in bladder cancer, offer a more complete explanation of the response of bladder cancer to immunotherapy, and suggest novel prognostic biomarkers to guide more effective immunotherapeutic strategies for bladder cancer.

## Materials and Methods

### Bladder Cancer Dataset Source and Preprocessing

In this study, we analyzed eight cohorts with a total of 1,482 bladder cancer patients: GSE31684, GSE32548, GSE32894, GSE69795, GSE83586, GSE86411, GSE87304, and GSE120736. All gene expression data sets were subjected to log2 transformation and quantile normalization. Batch effects from non-biological technical biases were corrected using the “ComBat” algorithm of the Sva package.

The gene expression data of 407 patients with bladder cancer were obtained from Genome Data Commons (https://portal.gdc.cancer.gov) as a validation set (**Supplementary Table 1**). Then, fragments per kilobase of exon model per million reads mapped values were transformed into transcripts per kilobase million values. IMvigor 210 cohort (NCT02108652) is a multicenter, single-arm phase II clinical study for evaluating the safety and efficacy of Tecentriq, a PD-L1 inhibitor, in patients with advanced urothelial carcinoma (15, 16). We obtained the corresponding clinical data and somatic mutation and copy number data based on the Creative Commons 3.0 License. The complete expression data, detailed clinical annotations (**Supplementary Table 2**), and somatic mutation data were obtained from IMvigor 210 Core Biologies (http://research-pub.gene.com/IMvigor210CoreBiologies), a complete documentation software and data package for the R statistical computing environment.

### Estimation of TME Cell Infiltration

Yi Xiao (17) found that a gene set that marks each TME infiltrating IC type contains 24 human IC subtypes. We used the single-sample gene set enrichment analysis (ssGSEA) algorithm to analyze the TME (**Supplementary Table 3**). The relative abundance of each cell infiltration was quantified. In addition, we also used the ESTIMATE algorithm (18) to estimate the ratio of the immune stromal components in the TME in each sample to further explore the differences in the TME scores among the different immunotypes, including ICs and stromal cells using ImmuneScore and StromalScore (**Supplementary Table 4**). A higher ImmuneScore or StromalScore indicates more immune or matrix components in the TME.

### Construction of Molecular Types Based on the Infiltration Level of 24 ICs in the TME

We used 407 samples from eight GEO datasets of TCGA-BLCA as the training set. Simultaneously, we included a total of 1,482 bladder cancer patients into the meta-cohort as the validation set. By analyzing the infiltration levels of 24 ICs and using the consensus clustering algorithm, we determined that the optimal clustering number (k value) of the two cohorts was 3. We used unsupervised cluster analysis (K-Means based on Euclidean distance) (19) to identify three different types. We used the ConsensusClusterPlus R package to perform the above steps, and performed 1000 repetitions (50 iterations with a resampling rate of 80%) to ensure the stability of the classification (20).

To further interpret the different characteristics of the three different immune subtypes, we compared them with the published classifications of several common bladder cancer subtypes in detail. The Baylor subtype established by Mo et al. (21) divided muscle-invasive bladder cancer (MIBC) and non-muscle-invasive bladder cancer into two subgroups each: basal and differentiated. Damrauer et al. (22) performed a consensus cluster analysis on the dataset, identified basal and luminal subtypes, and further identified 47 genes as subtype predictors in UNC. To perform unsupervised cluster analysis on 73 MIBC specimens, MDA subtype (23) with 2,252 genes (2,697 probes) was used, these genes produced three subtypes, namely, p53-like, luminal, and basal. Lund et al. (24) presented a six-class system based on global mRNA expression: urothelial-like, genomically unstable, epithelial infiltrated, squamous carcinoma (SCC)-like/mesenchymal (Mes)-like, SCC-like/urobasal B, and small-cell/neuroendocrine-like. CIT (25) used 2,707 genes to perform unsupervised cluster analysis on 129 MIBC patient samples and divided the patients into four subtypes (I, II, III, and IV) (26). These classifiers were combined into an R package (BLCAsubtyping (27); https://github.com/cit-bioinfo/BLCAsubtyping) and applied independently to the GEO and TCGA-BLCA datasets and the IMvigor 210 cohort.

### Identification of Differentially Expressed Genes (DEGs) Among Immunophenotypes and Signal Pathway Enrichment Analysis

The Bayesian method of the limma R package (28) was used to analyze the difference between the two groups. The DEGs were analyzed using gene ontology and Kyoto Encyclopedia of Genes and Genomes (KEGG) with the corrected *P* value < 0.05 and the absolute value of log fold change > 1 as the criteria to determine the significance of DEGs. GSEA was used to evaluate the skewness of the two distributions of the selected gene sets in the gene list sorted by a specific phenotype. The analyzed gene set was obtained from the Hallmark gene sets from the Molecular Signatures Database (MSigDB) (h.all.v7.1.entrez.gmt) using the clusterProfiler R package (29). We used the GSVA R package to perform Gene Set Variation Analysis (GSVA) for the assessment of gene enrichment to study the differences in biological processes among the three immune subtypes. GSVA is a non-parametric, unsupervised method used to estimate changes in pathway and biological processes in expression data set samples. We obtained the gene set “c5.all.v6.2.symbols.gmt” and 50 common biological pathway gene sets for GSVA analysis from the MSigDB database. We used the ssGSEA method to generate enrichment scores of these gene sets for each pathway in each sample, and compared the three immunotypes using the ssGSEA score of the pathways.

### Correlation Analysis With Core Biological Pathways of Bladder Cancer

We referred to a gene set related to certain biological processes provided by Mariathasan et al. (16) that included (1) immune checkpoint (2) antigen-processing machinery, (3) CD8 T-effector signature, (4) epithelial-mesenchymal transition (EMT) markers including EMT1, EMT2, and EMT3, (5) angiogenesis signature, (7) pan-fibroblast transforming growth factor (TGF)-β response signature, (8) Wnt targets, (9) DNA damage repair, (10) mismatch repair, (11) nucleotide excision repair, (12) DNA replication, and (13) antigen processing and presentation. Simultaneously, we obtained the Hallmark gene set from the MSigDB database and quantified the scores of 50 signal pathways using the ssGSEA algorithm to further reveal the differences in biological pathways related to different immunophenotypes. In addition, a set of genes associated with the differentiation of bladder cancer was obtained from the supplementary information by Kamoun et al. (27, 30).

### Correlation With Cancer-Immunity Cycle

The cancer-immunity cycle is an important framework for tumor immunotherapy research. It describes a cyclical process involving the immune system to eradicate cancer, which mainly includes seven steps: (1) cancer antigen release, (2) cancer antigen presentation, (3) initiation and activation, (4) T-cell transport to the tumor, (5) T cells penetration into the tumor, (6) T-cell recognition of cancer cells, and (7) T cell killing of cancer cells (31). The genetic information from each step were obtained from Tracking Tumor Immunephenotype (http://biocc.hrbmu.edu.cn/TIP/index.jsp). We quantified the scores of the seven steps using the ssGSEA algorithm, and compared the differences in the three scores to the seven steps in immunophenotyping.

### Analysis of Mutations and Copy Number Differences

The waterfall chart of the maftools software package was used to show the top 20 genes with high mutation frequency in the TCGA-BLCA cohort. For copy number analysis, GISTIC2.0 was used to identify significantly amplified or missing genomes (32). The burden of copy number loss or gain was calculated as the total number of genes with copy number changes at the focal and arm levels.

### The Construction and Evaluation of ICscore

By analyzing the level of 24 IC infiltration in each sample, we used the PCA method to construct a scoring system to evaluate the level of IC infiltration in a single patient with bladder cancer, and termed it ICscore. We tested the distribution differences of immune scores in different immune subtypes. We further explored and evaluated its predictive effects on the prognosis of patients with bladder cancer and their response to immunotherapy.

### Correlation Analysis With ICIS Response

To explore the predictive effect of our immunophenotyping on anti-PD-L1 immunotherapy, we used the IMvigor 210 cohort. In this study, we classified all or a portion of the responders as responders, and classified patients with stable disease or progressive disease as non-responders. We used the TIDE algorithm to evaluate the potential response to immune checkpoint blockade (ICB) treatment. The TIDE algorithm is a calculation method that uses gene expression profiles to predict the ICB response. It evaluates two different tumor-immune escape mechanisms, including the dysfunction of tumor-infiltrating cytotoxic T lymphocytes (CTLs) and the rejection of CTLs by immunosuppressive factors. The TIDE score can be a good assessment of the efficacy of anti-PD-1 and anti-CTLA4 treatments (33). Patients with a higher TIDE score have a higher chance of anti-tumor-immune escape and thus show a lower ICB treatment response rate. SubMAP (34) is used to compare the similarity of expression profiles and reflects the response to treatment. We used the SubMAP algorithm to predict the possibility of high and low ICscore of anti-PD1 and anti-CTLA4 response immunotherapy. The related annotation data were obtained from the research supplementary materials of Lu et al. (35).

### Statistical Analysis

The comparison between the two groups of normally distributed variables was performed using unpaired t- and Mann-Whitney U tests (also called Wilcoxon rank sum test) for non-normally distributed variables. The Kruskal-Wallis test and one-way analysis of variance were used as non-parametric and parametric analytical methods for comparison between the two groups, respectively. The differences between the groups of categorical variables were evaluated using the chi-squared test. The correlation coefficient between the two variables was calculated using Spearman and distance correlation analyses. The survminer R package was used to determine the demarcation point of each data set of each subgroup. The surv-cutpoint function was used to dichotomize the ICscore, and then the patients were divided into high and low subgroups according to the largest selected log-rank statistic to reduce the calculated batch effect. The Kaplan-Meier method was used to draw the survival curve of prognosis analysis, and the log-rank test was used to determine the significance of the difference. Univariate Cox regression model was used to calculate the hazard ratio (HR) of the ICscore, and a multivariate Cox regression model was used to determine the independent prognosis factor. Statistical analysis was performed on R software, with a P value < 0.05 (two-tailed) indicating significant significance.

## Results

### Identification of Three Different Immune Subtypes in Bladder Cancer

**Figure 1:**
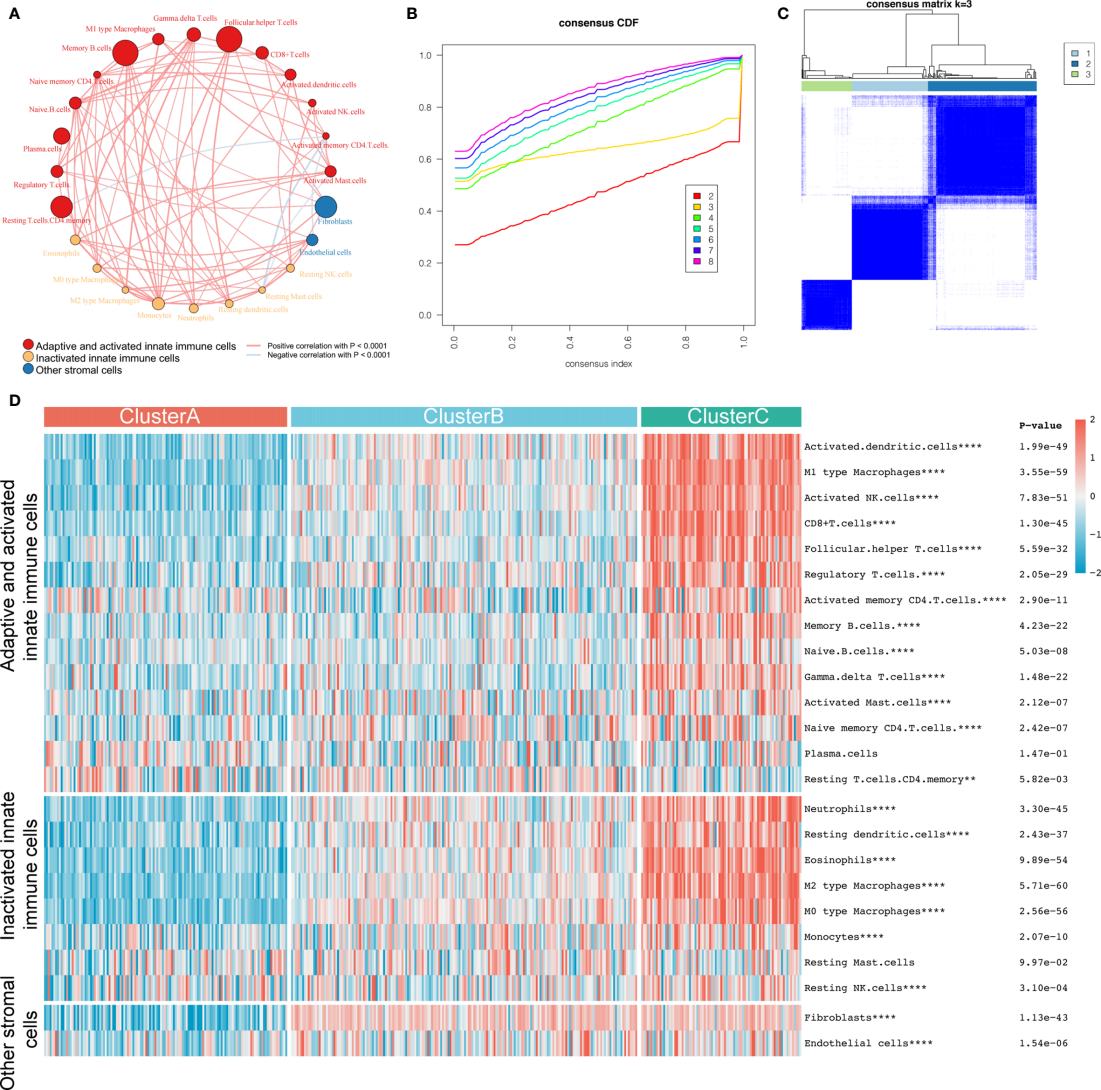
The construction of three different immune subtypes through k-means clustering. **(A)** We divided 24 types of immune cells into three subtypes (red: adaptive and activated innate immune cells; yellow: inactivated innate immune cells; blue: other stromal cells). The line connecting two cells represents the interaction between these factors. Red indicates positive correlation and light blue indicates negative correlation. The size of the circle symbolizes the *p* value (expressed as -log10 value) of the log-rank test. Impact of immune cells on bladder cancer overall survival (OS). The dot in the middle of each circle represents the influence of immune cells on the OS of bladder cancer. Green is a favorable factor and black is a risk factor. **(B, C)** We evaluated the stability of the clusters using the ConsensusClusterPlus R package and determined that the optimal number of clusters was 3. **(D)** All samples were divided into three clusters using the unsupervised clustering method: red, cluster A; light blue, cluster B; light green, cluster C. The heat map shows the infiltration level of the 24 immune cells in the three clusters. Red represents high infiltration and blue-green represents low infiltration. **P < 0.01; ****P < 0.0001.

We included 24 IC subtypes that are divided into three categories: adaptive and activated innate immune cells, inactivated innate immune cells, and other stromal cells. We drew the correlation network diagram of 24 ICs in bladder cancer (**Figure 1A**), and observed IC interaction and its significance in the prognosis of patients with bladder cancer. We found that most cells had a significantly positive correlation with the level of infiltration, while a few had a negative correlation, including activated memory CD4 T cells and resting T cells, memory CD4 T cells, resting mast and endothelial cells, and activated mast cells and resting mast cells. To select the optimal number of clusters, we analyzed 407 sample data from TCGA-BLCA to evaluate the stability of the clusters using the ConsensusClusterPlus package, and determined the optimal number of clusters to be 3 (**Figures 1B, C**). Then, we performed unsupervised clustering of the aforementioned bladder cancer samples and divided them into three clusters: A, B, and C. We found that most ICs were highly infiltrated in cluster C with the lowest infiltration in cluster A (**Figure 1D** and **Supplementary Figure 1**). The clustering results are in line with the immunological principles reported in a previous article: clusters A and B were similar to cold tumors, but they had different microenvironment composition phenotypes, and cluster C was a similar hot tumor. Eight meta-cohorts (GSE57303, GSE34942, GSE84437, ACRG/GSE62254, GSE15459, GSE29272, and TCGA-stomach adenocarcinoma) were used to verify repeatability (**Supplementary Figures 2A, B**), and we obtained almost the same clustering results.

### Correlation Analysis of Immunophenotyping With Clinical Features and Common Molecular Typing of Bladder Cancer

**Figure 2:**
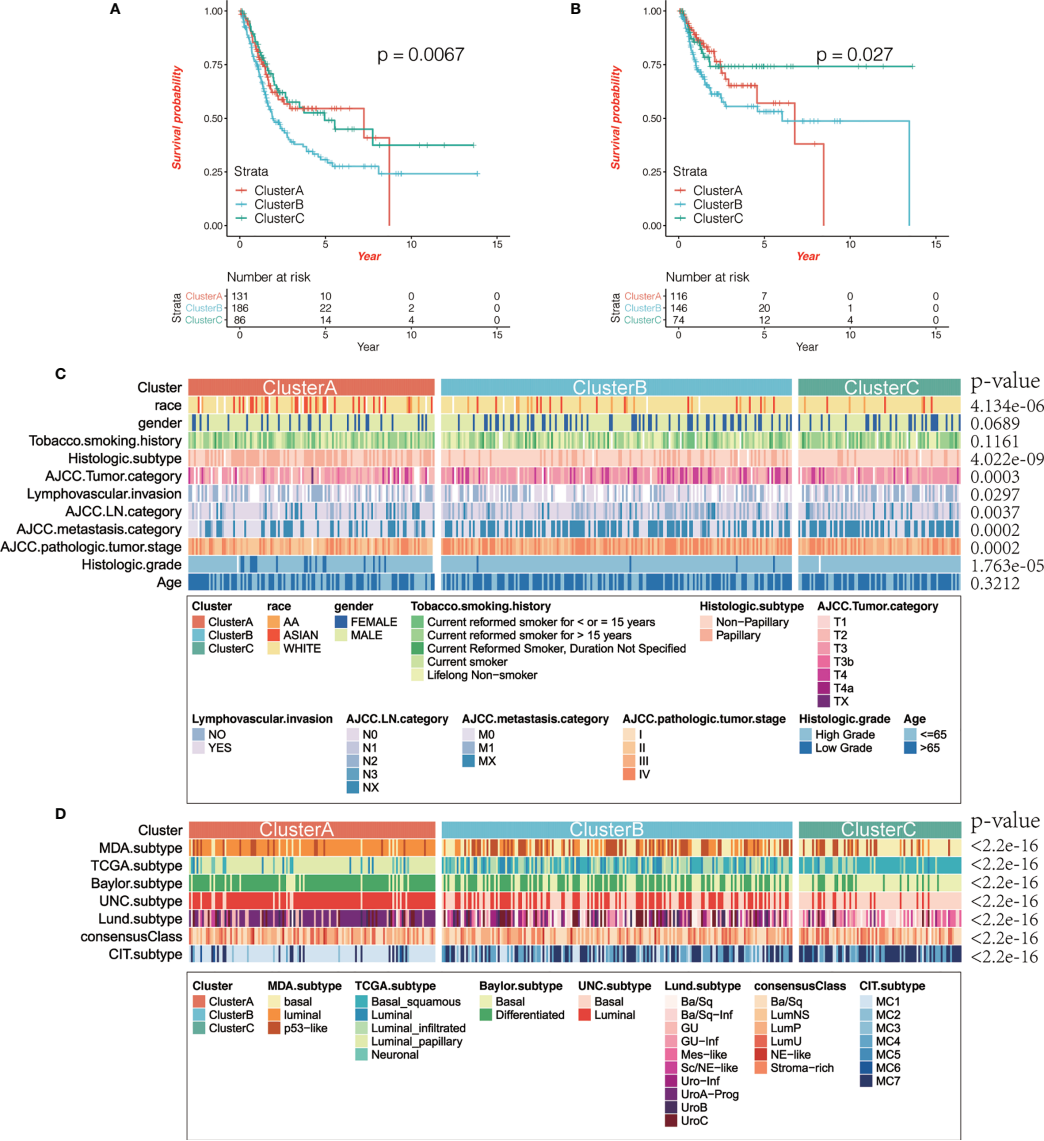
The correlation between immunophenotyping and clinical features. **(A, B)** Kaplan-Meier curve shows the prognostic significance of different immunotypes for overall survival (OS; A) and relapse-free survival (RFS; B). Compared with other subtypes, cluster B had the worst prognosis, while clusters A and C had better prognosis (log-rank test, OS: *P* = 0.0076, RFS: *P* = 0.027). **(C)** We analyzed the correlation between immunophenotyping and clinical features of bladder cancer (the *p* value is the result of chi-squared test). **(D)** We analyzed the correlation between immunophenotyping and published molecular typing of bladder cancer (the *p* value is the result of chi-squared test).

Based on the OS data from TCGA, we performed survival analysis on the TME phenotype and found that cluster B had the worst prognosis (log-rank test, *P* = 0.0076; **Figure 2A**) while clusters A and C showed better survival, and the relapse-free survival (RFS) results were similar to the OS results (log-rank test, *P* = 0.027; **Figure 2B**).

Then we compared the clinical characteristics of different immunotypes and displayed them through heat maps, and found that there was little difference in gender and age between the three groups (χ2 test, p>0.05), but there are significant differences in race, smoking time, TNM and tumor grade status (χ2 test, p<0.05) (**Figure 2C**). We compared and analyzed the immunophenotyping of several published subtypes of common bladder cancers. Compared with the Baylor subtype, cluster A had lesser basal subtypes with the differentiated subtype being predominant, while cluster C was the opposite. Compared with the UNC subtype, luminal subtype was predominant in cluster A, while basal subtype was predominant in cluster C. Compared with the CIT subtype, MC1 subtype was predominant in cluster A, while MC7 subtype was predominant in cluster C. Compared with the Lund subtype, urothelial-like A (UroA-Prog) was predominant in cluster A, while basal/squamous (Ba/Sq)-Inf, and Mes-like were the majority in cluster C. Compared with the MDA subtype, luminal subtype was predominant in cluster A, while the basal subtype was predominant in cluster C. Compared with the TCGA subtype, luminal papillary accounted for the vast majority in cluster A, and Ba/Sq was predominant in cluster C (χ2 test, all *P* values < 2.2e-16; **Figure 2D**).

### Analysis of the Differences Among the Three Immunophenotyping Signal Pathways

**Figure 3:**
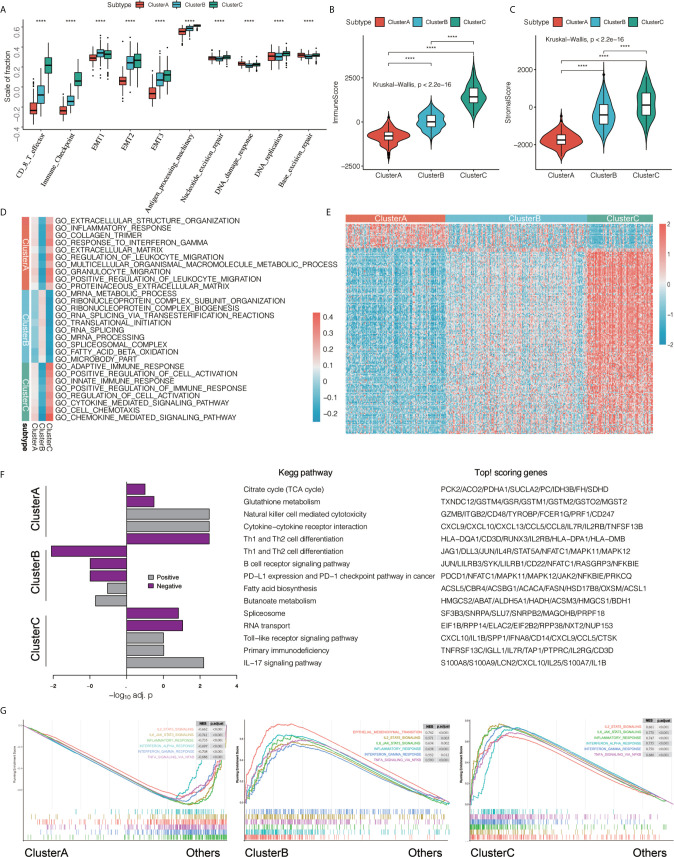
Features of three types of transcriptomes. **(A)** We compared the statistical differences in the scores of important biological gene sets using the Kruskal-Wallis test. ****P < 0.0001. **(B, C)** Using the ESTIMATE algorithm, we defined ImmuneScore and StromalScore. Using the Kruskal-Wallis test, we compared the differences in immune and matrix components in different immunotypes of tumor microenvironment, ****P < 0.0001. **(D)** Gene Set Variation Analysis (GSVA) shows the activation status of biological pathways in different immunophenotypes. Heat maps were used to visualize these biological processes. Red represents activated pathways and blue represents inhibited pathways. **(E)** We found differentially expressed genes that are related to a subtype by comparing each subtype with the others using the limma package. In the heat map visualization, red represents upregulation, and blue represents downregulation. **(F)** performs KEGG pathway enrichment on the characteristic genes of ClusterA and ClusterC, and uses a histogram for display. **(G)** GSEA enrichment analysis shows the enrichment status of biological pathways in different immunophenotyping.

We first compared the differences in the scores of important biological gene sets of bladder cancer among the three immunotypes. We found that CD8 T effector, immune checkpoint, EMT, and antigen processing machinery were significantly higher in cluster A and lowest in cluster C, while nucleotide excision repair, DNA damage response, DNA replication, and base excision repair were the lowest in cluster B (**Figure 3A** and **Supplementary Table 5**). In addition, we used the ESTIMATE algorithm to obtain the ImmuneScore and StromalScore, and compared the differences in immune and matrix components among the different immunotype TMEs. We found that ImmuneScore and StromalScore were the highest in cluster C, followed by cluster B, with cluster A having the lowest ImmuneScore and StromalScore (**Figures 3B, C**).

To further explore the differences in biological behavior among the three different immunophenotypes, we first performed GSVA enrichment analysis. Cluster A was significantly enriched in immunosuppressive signaling pathways such as extracellular structure organization, inflammatory response, collagen trimer, response to interferon γ, extracellular matrix, regulation of leukocyte migration, multicellular organismal macromolecule metabolic process, granulocyte migration, positive regulation of leukocyte migration, and proteinaceous extracellular matrix. Cluster B was enriched in mRNA metabolic process, ribonucleoprotein complex subunit organization, ribonucleoprotein complex biogenesis, RNA splicing *via* transesterification reactions, translational initiation, RNA splicing, mRNA processing, spliceosomal complex, fatty acid beta oxidation, and microbody part. However, cluster C was significantly related to biological processes related to immune activation, such as adaptive immune response, positive regulation of cell activation, innate immune response, positive regulation of immune response, regulation of cell activation, cytokine-mediated signaling pathway, cell chemotaxis, and chemokine-mediated signaling pathway (**Figure 3D**). We used the limma package to determine DEGs related to immunophenotyping (**Figure 3E** and **Supplementary Table 6**) and performed KEGG signaling pathway enrichment analysis (**Figure 3F** and **Supplementary Table 7**). We found that cluster A were mainly enriched in Citrate cycle (TCA cycle), Glutathione metabolism, Natural killer cell mediated cytotoxicity, Cytokine−cytokine receptor interaction, and Th1 and Th2 cell differentiation compared with the other two groups. However, cluster C were mainly related to B cell receptor signaling pathway, PD−L1 expression and PD−1 checkpoint pathway in cancer, Fatty acid biosynthesis, Butanoate metabolism. Cluster C were mainly related to Spliceosome, Toll−like receptor signaling pathway, Primary immunodeficiency, and IL-17 signaling pathway compared with the other two groups. Finally, we verified the differences in pathway enrichment among the three immunotypes using GSEA. We found that most of the immune signaling pathways were activated in clusters B and C, while immune activation-related biological pathways in cluster A were in an inhibited state (**Figure 3G**).

### The Differences in Expression of Immune Cycle Activation, Tumor Immunogenicity, and Immune Checkpoint Molecules in the Three Immunotypes

**Figure 4:**
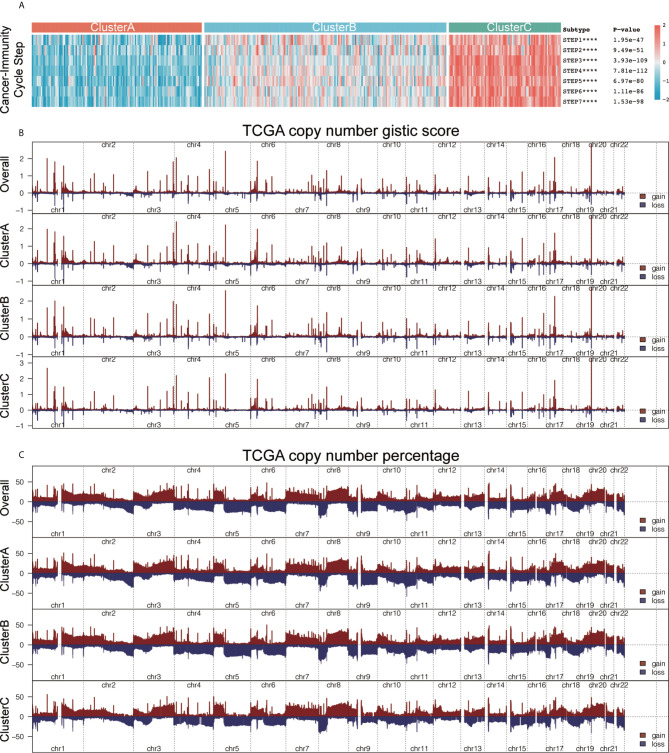
he characteristics of immunogenicity differences and genome groups of the three immunophenotypes. **(A)** Using the single-sample gene set enrichment analysis (ssGSEA) algorithm, we calculated the scores of the seven steps of the tumor-immune cycle. Using the Kruskal-Wallis test, we compared the statistical differences in the scores of the seven steps among the three immunotypes. ****P < 0.0001. **(B)** The plot illustrates the copy number GISTIC score of the gain (dark red) and loss (dark blue) of each gene in each cluster. **(C)**. The plot illustrates the frequency of the gain (dark red) and loss (dark blue) of each gene in each cluster. Copy number profiles for each cluster, with gains in dark red and losses in midnight blue. Gene segments are placed according to their location on chromosomes, ranging from chromosomes 1 to 22.

An anti-tumor-immune response must initiate a series of step-by-step events to effectively kill cancer cells. These steps are crucial to the cancer-immunity cycle. The dead tumor cells release antigens (step 1). The antigen and the major histocompatibility complex (MHC) complex on the surface of the antigen-presenting cells (APC) such as dendritic cells (a type of professional APC) form an antigen peptide-MHC complex (step 2). The T-cell receptor recognizes the binding between the antigen peptide-MHC complex on the surface of APC, the B7 molecule on the surface of APC and the dimer molecule CD28 on the surface of T cells, and the dual signal starts to activate T cells (the dual signal system regulation can review the immune response and tumor immunotherapy) (Step 3). Among them, CTLs are transported to the tumor tissue through blood circulation (step 4). CTLs enter the tumor tissue (step 5) and recognize tumor cells; (step 6) CTLs kill tumor cells (step 7), and release additional tumor-associated antigens (step 1 again). We obtained the important regulatory genes of each step and calculated the score of each step using the ssGSEA algorithm. We then explored the differences in the scores of the seven steps among the three immunotypes, and the results showed that the seven steps in cluster C had the highest score, followed by cluster B with cluster A having the lowest score (all *P* < 0.05; **Figure 4A**). We found that there were significant differences in genes associated with epithelial differentiation among different subtypes of bladder cancer. The expression of these genes was highest in cluster A, and the lowest in cluster C (**Supplementary Figure 3A**). We further studied the potential intrinsic immune escape mechanism of bladder cancer. Innate immune escape indicates that tumor cells directly mediate their own immune escape. The inherent immune escape has at least two aspects: the immunogenicity of the tumor and the expression of immune checkpoint molecules. Both subtypes had lower expression of MHC I-related antigen-presenting molecules than cluster C (all *P* < 0.001; **Supplementary Figure 3B**, top), and their immunogenicity was low. Overall, the tumor immunogenicity of cluster C was relatively high, while that of clusters A and B was relatively low. We demonstrated that cluster C had higher expression of costimulatory molecules (most *P* < 0.05) and immune checkpoint molecules compared with other clusters (most *P* < 0.05; **Supplementary Figure 3B**, bottom). We further demonstrated that immune infiltration is positively correlated with immunogenicity and the expression of most checkpoint molecules. We obtained the gene set “c5.all.v6.2.symbols.gmt” and 50 common biological pathway gene sets for GSVA analysis from the MSigDB database. We used the ssGSEA method to generate enrichment scores of these gene sets for each pathway in each sample, and compared the three immunotypes using the ssGSEA score of the pathways (**Supplementary Figure 3C**).

### The Characteristics of the Tumor Somatic Mutation and Immune Subtype Copy Number

We explored the underlying mechanisms leading to the formation of these microenvironmental phenotypes, aiming to identify potential targets to reverse the formation of low immune infiltration. We used the maftools package to analyze the distribution of somatic mutations and the difference in tumor mutational burden (TMB) among the three subtypes (**Supplementary Figure 3D**). Clinical trials and preclinical studies have showed that when ICB therapy is used, higher somatic TMB is associated with enhanced response, long-term survival, and lasting clinical benefits in patients. A single altered gene can mediate resistance or sensitivity to immunotherapy. In addition, we also analyzed the copy number characteristics of the different immune subtypes (**Supplementary Figure 3E**) and found three different immunotype composite copy number profiles, including GISTIC score and percentage/frequency (**Figure 4B**). Our analysis shows that there are certain genomic changes in different immune subtypes, which may be the cause of the difference in immunotyping.

### ICscore Is a Reliable and Independent Prognostic Biomarker for Evaluating Bladder Cancer Patients

**Figure 5:**
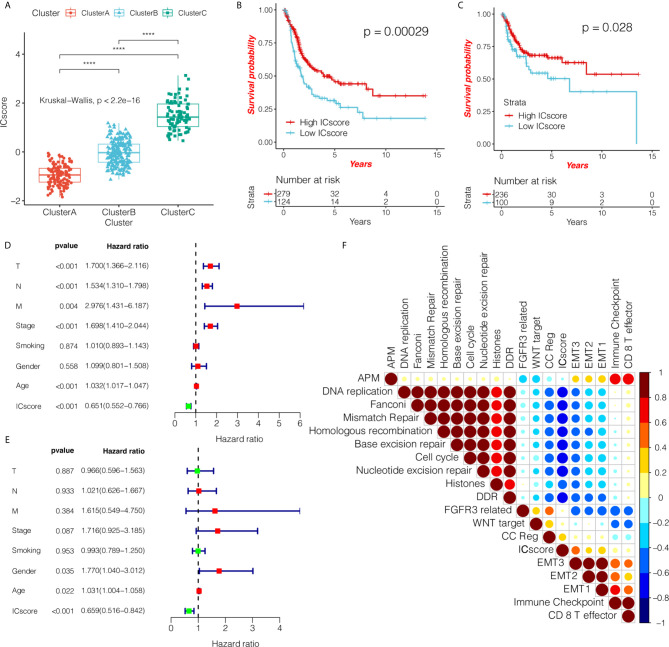
ICscore is a reliable and independent prognostic biomarker for evaluating bladder cancer patients. **(A)** The box plot shows the difference in ICscore of the three immunotypes in The Cancer Genome Atlas (TCGA) cohort. Cluster C had the highest ICscore, followed by cluster B, with cluster A having the lowest score. The statistical difference among the three groups was compared using the Kruskal-Wallis test. The Kaplan-Meier curve shows the prognostic significance of ICscore in the TCGA cohort for overall survival **(B)** and RFS – relapse-free survival. **(C)** Patients with high ICscore showed significant survival benefits (hazard ratio (HR) = 0.65, 95% confidence interval (CI): 0.55–0.77; HR = 0.8, 95% CI: 0.65–0.99). **(D, E)** Using univariate and multivariate Cox regression analyses, we determined that ICscore is a reliable and independent prognostic biomarker for patients with bladder cancer. The length of the horizontal line represents the 95% CI for each group. The vertical dotted line indicates HR = 1. The HR value (HR<1) indicates that a high ICscore is a favorable prognostic biomarker. **(F)** Using Spearman analysis, we clarified the correlation between ICscore and known gene markers in the TCGA cohort. Negative correlation is marked in blue, and a positive correlation is marked in red. ****P < 0.0001.

Considering the individual heterogeneity and complexity of the tumor-immune microenvironment, we constructed a scoring system to quantify the level of immune cell infiltration based on these immune cells in a single bladder cancer patient using the ICscore. We first analyzed the differences in ICscore among the three immunotypes, and found that the ICscore of cluster C was the highest, followed by cluster B, with cluster A having the lowest ICscore (**Figure 5A**). This result is in line with our previous trend of results. We further determined the value of ICscore in predicting patient prognosis. Patients were divided into low or high ICscore groups according to the survminer software package. The Kaplan-Meier curve showed that ICB treatment may be more beneficial in patients with higher ICscore, regardless of OS (**Figure 5B**; HR = 0.65, 95% confidence interval (CI): 0.55–0.77) or RFS (**Figure 5C**; HR = 0.8, 95% CI: 0.65–0.99). Simultaneously, we tested whether the ICscore could be used as an independent prognostic factor for bladder cancer. We used univariate and multivariate Cox regression models to analyze factors including patient age, sex, tumor, node, metastasis, smoking, and stage statuses, and confirmed that the ICscore is a reliable and independent prognostic biomarker for assessing patient prognosis (**Figures 5D, E**). We further explored the correlation between the ICscore and scores of important biological pathways, and found that the ICscore was negatively correlated with DNA replication, mismatch repair, homologous recombination, base excision repair, cell cycle, and nucleotide excision repair (**Figure 5F**).

### ICscore Is a Reliable Prognostic Biomarker and Predictor of Immune Checkpoint Inhibitor Response

Immunotherapy using anti-PD-1/PD-L1 and anti-CTLA4 has become a breakthrough in cancer treatment. We systematically studied whether our immune classification and ICscore can predict the patient response to ICB therapy.

**Figure 6:**
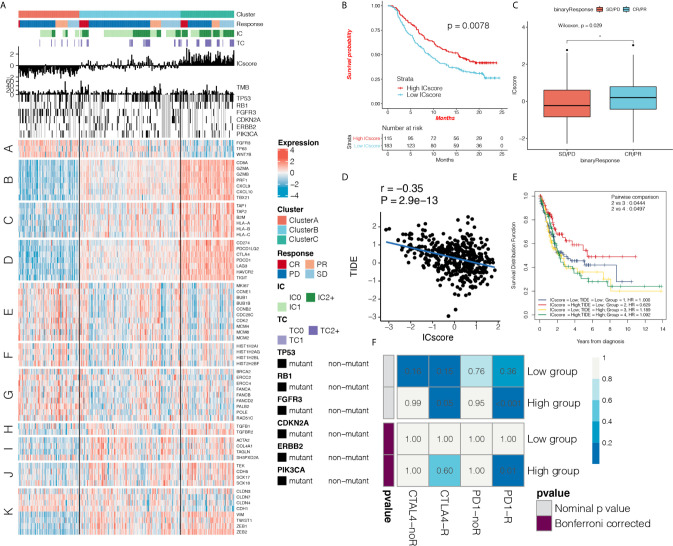
ICscore is a reliable prognostic biomarker and predictor of immune checkpoint inhibitor response. **(A)** Heat map shows the effect of immunophenotyping and ICscore on anti-programmed death-ligand 1 (PD-L1) treatment in IMvigor 210 cohort, IC: PD-L1 expression on cells, TC: PD-L1 expression on cells. A: *FGFR3* gene signature; B: CD8 T-effector signature; C: antigen-processing machinery; D: immune checkpoint signature; E: MKI67 and cell cycle genes; F: DNA replication-dependent histones; G: DNA damage repair genes; H: transforming growth factor (TGF)-β receptor and ligand; I: TGF-β response signal signature (*F-TBRS*) genes; J: EMT markers; K: angiogenesis signature. **(B)** Kaplan-Meier curve shows the prognostic significance of ICscore for patient survival in the IMvigor210 cohort. Patients with high ICscore showed significant survival benefits (hazard ratio (HR) = 0.65, 95% confidence interval (CI): 0.55–0.77). **(C)** Box plot shows the difference in ICscore between complete response (CR)/partial response (PR) group and stable disease/progressive disease group after receiving anti-PD-L1 immunotherapy. The ICscore of CR/PR group was higher than that of stable disease/progressive disease group (Wilcoxon test, *p* = 0.0029) **(D)** Using Spearman analysis, we clarified the correlation between ICscore and Tumor Immune Dysfunction and Exclusion (TIDE) in The Cancer Genome Atlas (TCGA) cohort. **(E)** Kaplan-Meier curve shows the prognostic significance of the combination of ICscore and TIDE in the TCGA cohort for patient survival. Patients with high ICscore + low TIDE score had the best prognosis. **(F)** Using the SubMAP algorithm, we inferred the possibility of anti-PD1 and anti- cytotoxic T-lymphocyte-associated protein 4 (CTLA4) response immunotherapy in the high and low ICscore groups. High ICscore group may respond better to PD-1 treatment (Bonferroni-corrected *P* = 0.01). *P < 0.05.

First, we verified the three immunotypes in bladder cancer (**Supplementary Figures 4A, B**) based on the IMvigor 210 cohort. Simultaneously, we confirmed that there were fewest patients with complete response (CR) in cluster A, and the levels of PD-L1 expression on ICs and tumor cells were significantly lower than those of the other two groups (**Figure 6A** and **Supplementary Figures 4C–F**). We used a composite heat map to show the differences in TMB and important mutation driver genes of bladder cancer among the three immunotypes (**Figure 6A**). We found that there was a significant increase in mutations in the fibroblast growth factor receptor 3 (*FGFR3*) gene in cluster A. In addition, gene sets were significantly upregulated in cluster A, such as *FGFR3* gene signature, *MKI67*, cell cycle genes, DNA replication-dependent histones, and DNA damage repair genes, while genes such as CD8 T-effector signature, antigen-processing machinery, immune checkpoint signature were inhibited in Cluster A (**Figure 6A** and **Supplementary Figure 4G**), which have been demonstrated to have an important effect on immunotherapy.

Based on the OS data from IMvigor 210 cohort, we performed survival analysis on the TME phenotype and found that cluster C showed better survival (**Supplementary Figure 5A**). The predictive effect of ICscore on anti-PD-L1 immunotherapy response was also systematically evaluated. In patients treated with anti-PD-L1, the Kaplan-Meier curve showed that a high ICscore had better prognosis for anti-PD-L1 therapy (HR = 0.85, 95% CI: 0.73–0.98; **Figure 6B**), and the ICscore of the CR/partial response (PR) group was higher than that of the stable disease/progressive disease group (Wilcoxon test, *p* = 0.0029; **Figure 6C**). We found that the ICscore was positively correlated with PD-L1 expression on ICs and tumor cells. It was confirmed that high ICscore are closely related to immune checkpoint expression (**Supplementary Figures 5B, C**). Comparison of the differences in the ICscore in immune subtypes revealed that ICscore was the lowest in desert tumors, moderate in excluded tumors, and highest in inflamed tumors (**Supplementary Figure 5D**). These results suggest that our immunophenotyping method and ICscore had a prominent predictive effect on anti-PD-L1 therapy.

The TIDE score integrates T-cell dysfunction and removal characteristics, and simulates tumor-immune escape at the level of tumor-infiltrating CTLs. Compared with other biomarkers, TIDE has an advantage in predicting the efficacy of anti-PD1 and anti-CTLA4 treatments. We further explored the correlation between the IC and TIDE scores. The ICscore was negatively correlated with TIDE (r = -0.35, *P* = 2.9e-13; **Figure 6D**). This again suggests that patients with high ICscore may show a better response to immunotherapy, and the combination of IC and TIDE scores can improve the prediction of patient prognosis (**Figure 6E**). We found that ICscore is negatively correlated with *IFNG* and *CD274* (r = -0.69, *P* = 4.6e-58; r = -0.70, *P* = 6.6e-62; **Supplementary Figures 5E, F**). Using the receiver operating characteristic (ROC) algorithm, we found that the ICscore (AUC 0.716, 95% CI: 0.647-0.787) can predict the responsiveness of anti-PD-L1 therapy well (area under the curve = 0.716), and compared with TMB (AUC 0.723, 95% CI: 0.643-0.793), tumor neoantigen burden(TNB, AUC 0.763, 95% CI: 0.688-0.829), *CD274* (AUC 0.723, 95% CI: 0.643-0.793) and *IFNG* (AUC 0.661, 95% CI: 0.574-0.735), the ICscore can equally predict the responsiveness of immunotherapy (P > 0.05; **Supplementary Figures 5G, H**), We used the SubMAP algorithm to predict the possibility of response to anti-PD1 and anti-CTLA4 immunotherapy in the high and low ICscore groups. We also confirmed that the group with a high ICscore may respond better to treatment (Bonferroni-corrected *P* = 0.01; **Figure 6F**).

## Discussion

We analyzed 1,889 bladder cancer samples using a series of unsupervised learning methods, explored the existence of three different microenvironmental phenotypes in bladder cancer, and verified its reproducibility. Our clustering results are in line with the immunological principles described in previous studies (36, 37). We found that cluster C had hot tumors with relatively high innate and adaptive IC infiltration while cluster A had immune desert type tumors (cold tumors), which was characterized by relatively low microenvironmental cell infiltration. In addition, our data clearly show that there were large differences in clinical characteristics among the different immune subtypes. Compared with other clusters, cluster B had poor prognosis in both OS and RFS, but there was not much of a difference in prognosis between clusters C and A. The difference in survival was consistent with previous studies.

In many solid tumors, although CTLs infiltrate the tumors to a greater extent, T-cell dysfunction is also strong, which may weaken the ability of CTLs to kill cancer cells and may promote the growth and progression of tumors, resulting in invasion and metastasis (33, 38). Compared with other common subtypes of bladder cancer, we found that cluster A is similar to luminal subtypes, while cluster C is similar to basal subtypes, squamous features are characteristic of basal bladder cancer. In addition, anti-CTLA4 and EGFR targeting drugs of T cell regulators may also have relatively high response in basal subtypes, which is partially consistent with our results (21–24).

We found that the poor prognosis of cluster C may be due to higher immunosuppression and lower immunoreactivity in the TME. Though cluster C had the highest immune cell infiltration, it exhibited a matrix-activated state with highly expressed EMT, TGF-β pathway, and angiogenesis, which are T-cell inhibitory (39, 40). This may be an important reason why the prognosis of cluster C is not as good as we predicted. EMT is a key process of cancer progression and metastasis, which is not only an important driving force of malignant tumor, but also essential for immune resistance. As a classic EMT driving molecule, TGF-β can change the cytotoxic effects including perforin, granzyme A, granzyme B, Fas ligand and IFN-γ (41, 42), therefore, targeting EMT may provide important insights for immunotherapy.

In addition, our results confirmed that there was a significantly high expression of the immune checkpoint molecules in cluster C. These results emphasize that though cluster C had more immune cells infiltrated in the tumors, its IC dysfunction and immune escape were also stronger, which may weaken the ability of ICs to kill cancer cells. This result is consistent with previous research reports, and is due to higher immunosuppression and lower immunoreactivity in the TME. Such patients are often more suitable for immune checkpoint inhibitor treatment (43, 44). Our research may also help promote research on the regulation mechanism of immune infiltration in bladder cancer. The transition from cold to hot tumors is currently being researched (45), and specific biological pathways may drive the formation of these microenvironmental phenotypes. We found that in cluster C RNA transport, spliceosome and other pathways were significantly enriched, while in cluster A, glutathione metabolism and citrate cycle (TCA cycle) were enriched, these results suggest that RNA processing and metabolism may play an important role in tumor immune microenvironment. It may provide direction for immunomodulation in the future.

In this study, differences in genes among different subtypes were shown to be significantly related to immune-related biological pathways. The differential genes obtained by comparing cluster A with the other two groups revealed that the characteristic genes related to cluster A were mainly enriched in signaling pathways such as cytokine-cytokine receptor interaction, Wnt signaling pathway, and Toll-like receptor signaling pathway. In addition, our GSEA and GSVA analyses revealed that many biological pathways related to immune activation of cluster A were significantly inhibited, while the signal pathways such as inflammatory response, response to interferon γ, and extracellular matrix were significantly activated. We confirmed that one of the most prominent biological characteristics was the significant change in *FGFR3*, including mutations and overexpression, and a significant increase in *FGFR3* gene mutations in cluster A. Previous studies have confirmed that *FGFR* oncogenic mutations occur in a fifth of bladder cancers (46, 47). The protein encoded by *FGFR3* is a member of the FGFR family and is related to Ras protein kinase/mitogen-activated protein kinase activation, angiogenesis inhibition, fibroblast activation, and EMT (47). We speculate that the low chemotaxis of innate ICs induced by FGFRs may be the cause of the poor immune permeability in cluster A. FGFR3 is a promising therapeutic target in multiple preclinical trials, but further studies are needed to verify the benefits of FGFR3 inhibitors in these subgroups. In addition, we have shown the copy number and mutation differences in different immune subtypes, providing evidence for exploring the reasons for the formation of immune subtypes at the genomic level. These results further reverse the poor infiltration characteristics of cells in the TME, and provide new ideas and targets for transforming cold tumors into hot tumors, which will help the development of new combinations of drugs or new immunotherapy drugs.

Our research may also guide more effective patient-specific immunotherapy strategies such as PD-1/PD-L1 therapy (48). However, its efficacy is high only in a small number of patients. Several studies have found the TMB are not effective biomarkers for predicting the benefits of ICB (38). Therefore, the establishment of predictive biomarkers for checkpoint immunotherapy is essential to maximize the benefits of treatment (49, 50).

Considering the heterogeneity and complexity of the individual TME, we established a scoring system to evaluate the level of IC infiltration in the TME. Univariate and multivariate Cox regression analyses showed that ICscore is a reliable and independent prognostic biomarker. We demonstrated the predictive value of the ICscore for the use of anti-PD-L1 drugs (atezolizumab) using the IMvigor 210 cohort. There was a significant difference in ICscore between non-responders and responders.

The TIDE score integrates T-cell dysfunction and removal characteristics, and simulates tumor-immune escape with different levels of tumor-infiltrating CTLs. Compared with other biomarkers, its advantages are very prominent (33, 51). A higher tumor TIDE score is related to a poorer efficacy of immune checkpoint suppression therapy. Our research confirmed that ICscore is negatively correlated with TIDE, which is consistent with our previous inference. In addition, the ICscore is negatively correlated with interferon γ and CD274, and these factors are T-cell inhibitory (17, 33). We could predict the possibility of anti-PD1 and anti-CTLA4 immunotherapy response in high and low ICscore groups using the SubMAP algorithm. This also confirmed that the high ICscore group may respond better to treatment.

Although important results were obtained in this study, there were some limitations. First, although our study included a large sample size of the bladder cancer cohort, sampling deviations caused by using different platforms may cause some subjectivity in gene expression values. At the same time, we need to pay attention to the potential caveat of bulk tumor RNAseq analysis (e.g. variable cellularity of different samples, contamination of lymph tissue in sample collection, etc). Second, our research provides new insights into the stromal microenvironment of bladder cancer and related treatment strategies. However, this study is limited by being prospective. Therefore, our findings should be further confirmed in clinical studies. In addition, immunogenomic analysis cannot reflect causality. The potential driving factors in our research, such as the FGFR3 pathway require further functional verification. This part of our study is currently undergoing experimental research and verification.

In conclusion, this study defined three heterogeneous bladder cancer microenvironmental phenotypes and illustrated their clinical significance.

We conducted a comprehensive analysis of the characteristics and differences of the three immunophenotypes. This will help elucidate the response of bladder tumors to immunotherapy and provide new strategies for cancer treatment. In addition, we identified a TME-based score that can effectively predict the survival outcome of bladder cancer patients. The TME score is a potential and powerful biomarker for the prognosis and clinical response evaluation of immunotherapy. Our findings provide novel ideas for improving the clinical response of bladder cancer patients to immunotherapy, identifying different tumor immunophenotypes, and promoting personalized cancer immunotherapy in the future.

## Data Availability Statement

The original contributions presented in the study are included in the article/**Supplementary Material**. Further inquiries can be directed to the corresponding author.

## Author Contributions

XC and KC designed the study. XC, HC, KC, and DH analyzed and interpreted the data. XC, YZ, and YC wrote this manuscript. MX, HL, and ZW edited and revised the manuscript. All authors contributed to the article and approved the submitted version.

## Funding

This work was supported by the National Natural Science Foundation of China (81874137), the science and technology innovation Program of Hunan  Province (2020RC4011),  the Outstanding Youth Foundation of Hunan Province (2018JJ1047), the Hunan Province Science and Technology Talent Promotion Project (2019TJ-Q10), Young Scholars of "Furong Scholar Program" in Hunan Province, and the Wisdom Accumulation and Talent Cultivation Project of the Third xiangya hosipital of Central South University (BJ202001).

## Conflict of Interest

The authors declare that the research was conducted in the absence of any commercial or financial relationships that could be constructed as a potential conflict of interest.

## Supplementary Material

Supplementary Figure 1 Landscape of the microenvironment phenotypes in The Cancer Genome Atlas Urothelial Bladder Carcinoma (TCGA-BLCA) data set. **(A)**. The difference in infiltration of 24 immune cells in the three immunotypes was compared using the Kruskal-Wallis test, and displayed by box plot. **P* < 0.05; ***P* < 0.01; ****P* < 0.001; *****P* < 0.0001.

Supplementary Figure 2 A meta-cohort consisting of a total of 1,482 bladder cancer patients with eight Gene Expression Omnibus (GEO)-independent data sets verified the repeatability of the clustering results. **(A, B)**. The cluster stability was evaluated using the ConsensusClusterPlus R package, and the optimal number of meta-cohort clusters was determined to be 3. **c**. All samples were divided into three clusters using the unsupervised clustering method, and representative colors were assigned. Red: cluster A, light blue: cluster B, light green: cluster C. The heat map shows 24 immune cell infiltration levels in the three clusters where red represents high infiltration and blue-green represents low infiltration.

Supplementary Figure 3 Signal pathway differences and copy number characteristics among the three immunophenotypes. **(A)**. The difference in Urothelial differentiation genes expression in the three immunotypes was compared using the Kruskal-Wallis test, and displayed by box plot. **P* < 0.05; ***P* < 0.01; ****P* < 0.001; *****P* < 0.0001. **(B)**. Using the Kruskal-Wallis test, we compared the differences in expression of related molecules of MHC I, MHC II, other MHC, co-inhibitors, and co-stimulators among three immunotypes. In the heat map, red represents upregulation and blue represents downregulation. **P* < 0.05; ***P* < 0.01; ****P* < 0.001; *****P* < 0.0001. **(C)**. The Kruskal-Wallis test was used to compare the score differences of 50 common biological gene sets in the three immunotypes and is displayed using a heat map. Red represents upregulation and blue represents downregulation. **P* < 0.05; ***P* < 0.01; ****P* < 0.001; *****P* < 0.0001. **(D)**. The figure shows the somatic mutations in the 20 genes with the highest frequency among the three immunophenotypes. Each column represents an individual patient. The upper panel shows tumor mutational burden, and the numbers on the right indicate the mutation frequency of each gene. We used different colors to represent different mutation types, and the small graph on the right shows the proportion of each mutation type. **(E)**. Detailed cytobands with focal amplification (top) and focal deletion (bottom) in the three groups generated using the GISTIC2.0 software. The q value of each locus is plotted horizontally.

Supplementary Figure 4 The correlation among the three immunotypes and their response to immunotherapy. **(A, B)**. The cluster stability was evaluated using the ConsensusClusterPlus R package, and the optimal number of clusters in the IMvigor210 cohort was determined to be 3. **(C–F)**. The stacked histogram shows the three immunotypes and anti-programmed death-ligand 1 (PD-L1) efficacy **(C)**, PD-L1 expression in immune cells **(D)**, PD-L1 expression in tumor cells **(E)**, and the defined immune subtype correlation **(F)**.

Supplementary Figure 5 ICscore is a reliable predictor of immune checkpoint inhibitor response. **(A)**. Kaplan-Meier curve shows the prognostic significance of different immunotypes for overall survival in IMvigor210 cohort (OS; A) **(B-D)**. The box plot shows the correlation between ICscore and programmed death-ligand 1 (PD-L1) expression in cells. **(A)**, PD-L1 expression in cells **(B)**, and the differences among the three defined immune subtypes. Kruskal-Wallis test was used to compare the differences between multiple groups, **P* < 0.05; ***P* < 0.01; ****P* < 0.001; *****P* < 0.0001. **(E, F)**. Spearman analyzed the correlation between ICscore, and CD274 and interferon γ in the IMvigor210 cohort. **(G, H)**. Receiver operating characteristic (ROC) curve identified that ICscore can predict the responsiveness of anti-PD-L1 therapy well (area under the curve = 0.716).

Supplementary Table 1 TCGA database clinical information of bladder cancer.

Supplementary Table 2 IMvigor210 cohort clinical information.

Supplementary Table 3 The level of immune cell infiltration in the sample calculated by ssGSEA.

Supplementary Table 4 TCGA sample ESTIMATEScore.

Supplementary Table 5 TCGA sample TMEScore

Supplementary Table 6 Comparison of genetic differences between subtypes by limma.

Supplementary Table 7 Exploring the enrichment of differential signaling pathways of each subtype through the method of GSEA.
