# Supplementary figures and images for: Analysis of Tumor Microenvironment Characteristics in Bladder Cancer: Implications for Immune Checkpoint Inhibitor Therapy
Source: Front Immunol. 2021 Apr 15;12:672158. doi: 10.3389/fimmu.2021.672158 (PMC8082152; doi:10.3389/fimmu.2021.672158)

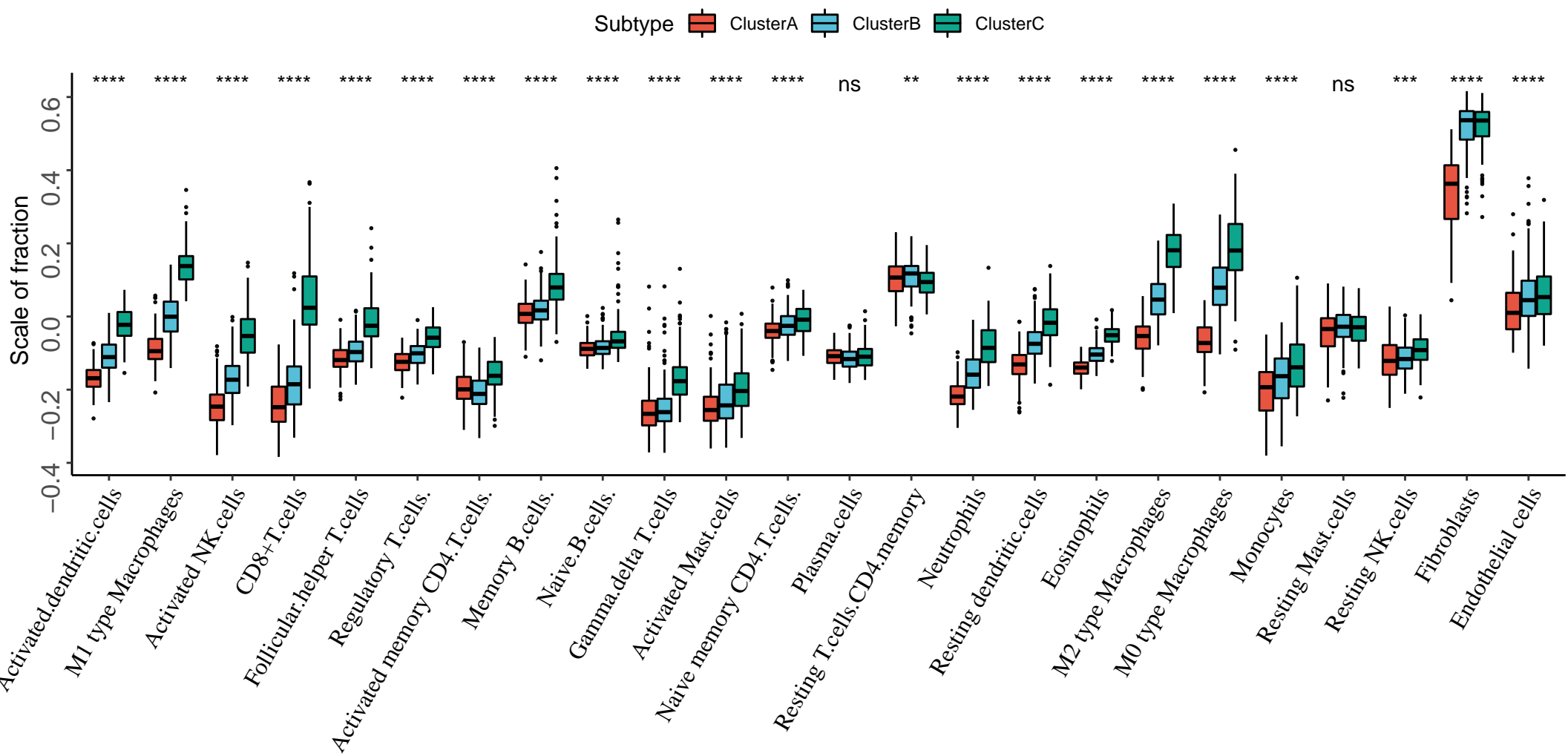

Supplement: Supplementary Figure 1 — Landscape of the microenvironment phenotypes in The Cancer Genome Atlas Urothelial Bladder Carcinoma (TCGA-BLCA) data set. (A). The difference in infiltration of 24 immune cells in the three immunotypes was compared using the Kruskal-Wallis test, and displayed by box plot. *P < 0.05; **P < 0.01; ***P < 0.001; ****P < 0.0001. [file Image_1.pdf]

A

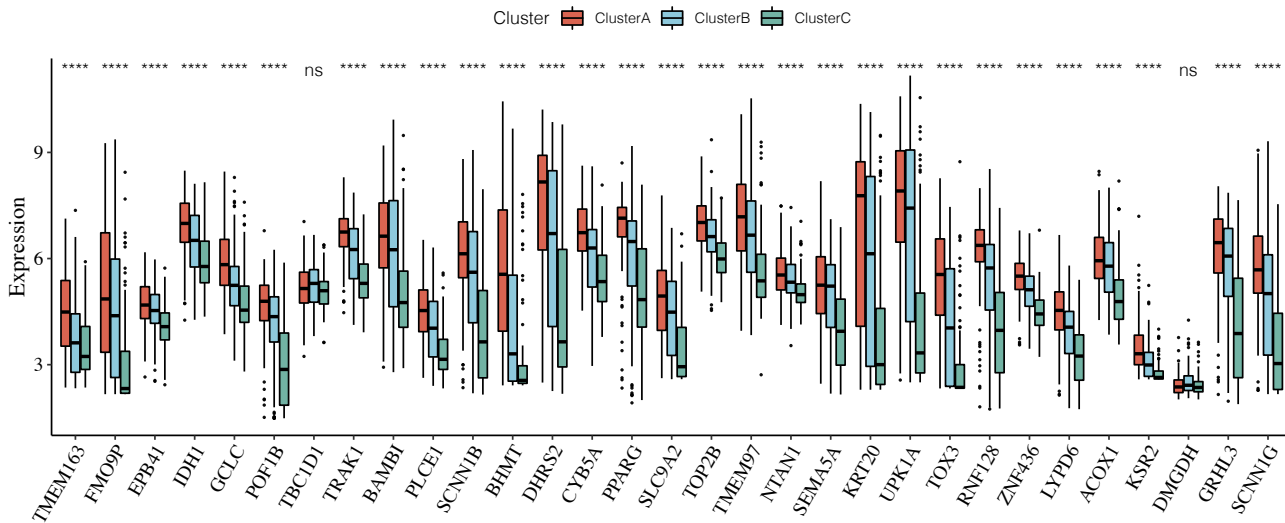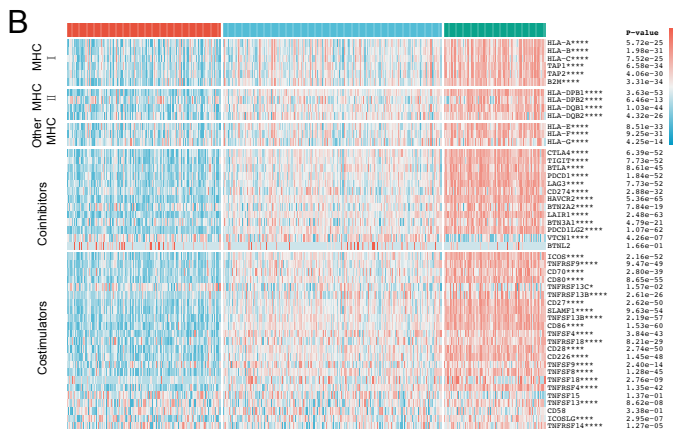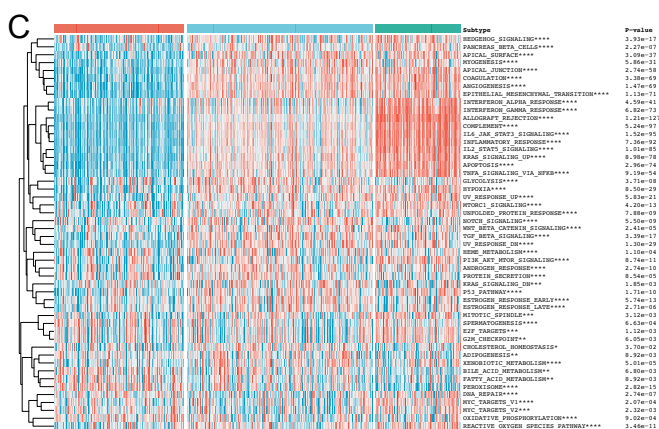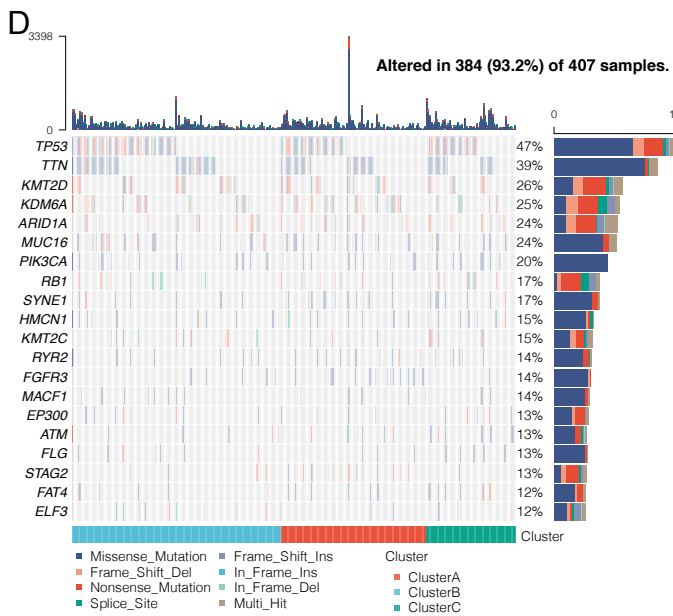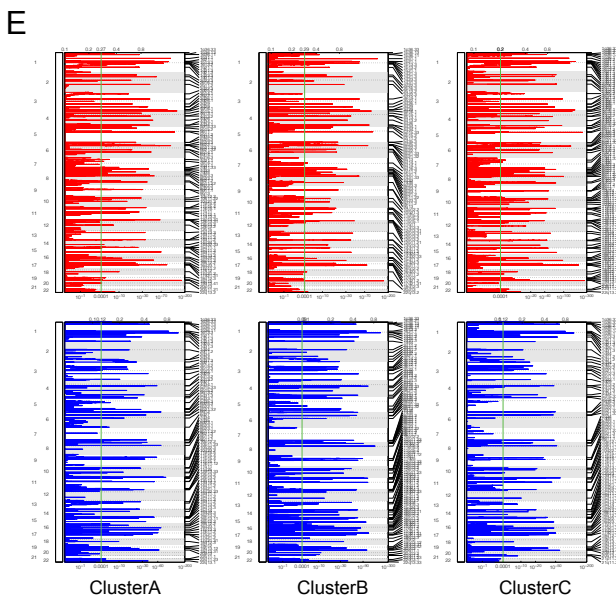

Supplement: Supplementary Figure 2 — A meta-cohort consisting of a total of 1,482 bladder cancer patients with eight Gene Expression Omnibus (GEO)-independent data sets verified the repeatability of the clustering results. (A, B). The cluster stability was evaluated using the ConsensusClusterPlus R package, and the optimal number of meta-cohort clusters was determined to be 3. c. All samples were divided into three clusters using the unsupervised clustering method, and representative colors were assigned. Red: cluster A, light blue: cluster B, light green: cluster C. The heat map shows 24 immune cell infiltration levels in the three clusters where red represents high infiltration and blue-green represents low infiltration. [file Image_2.pdf]

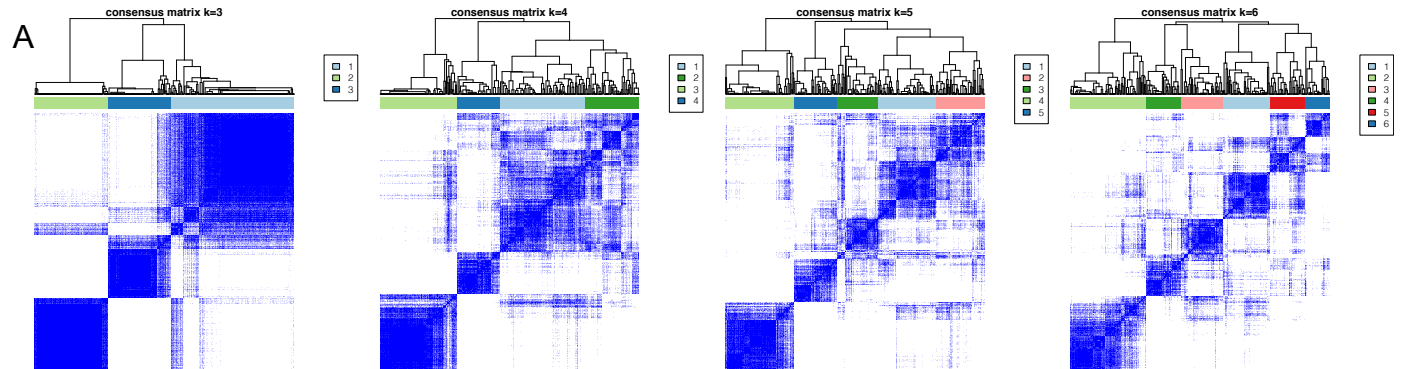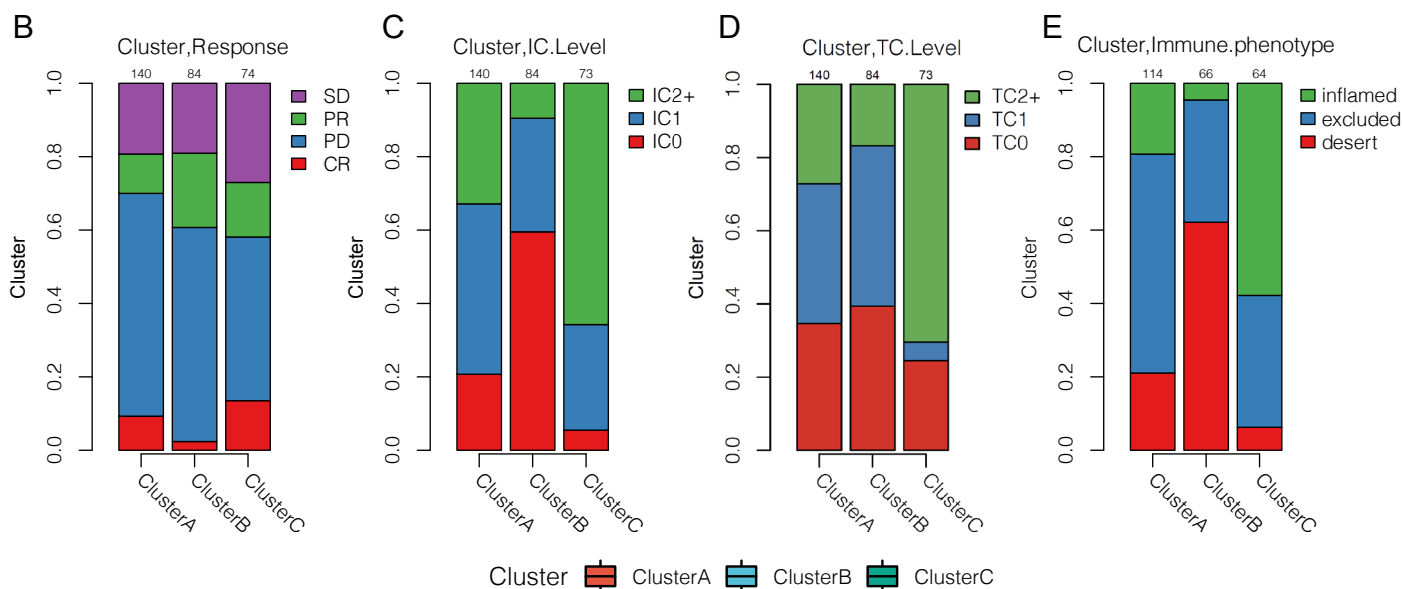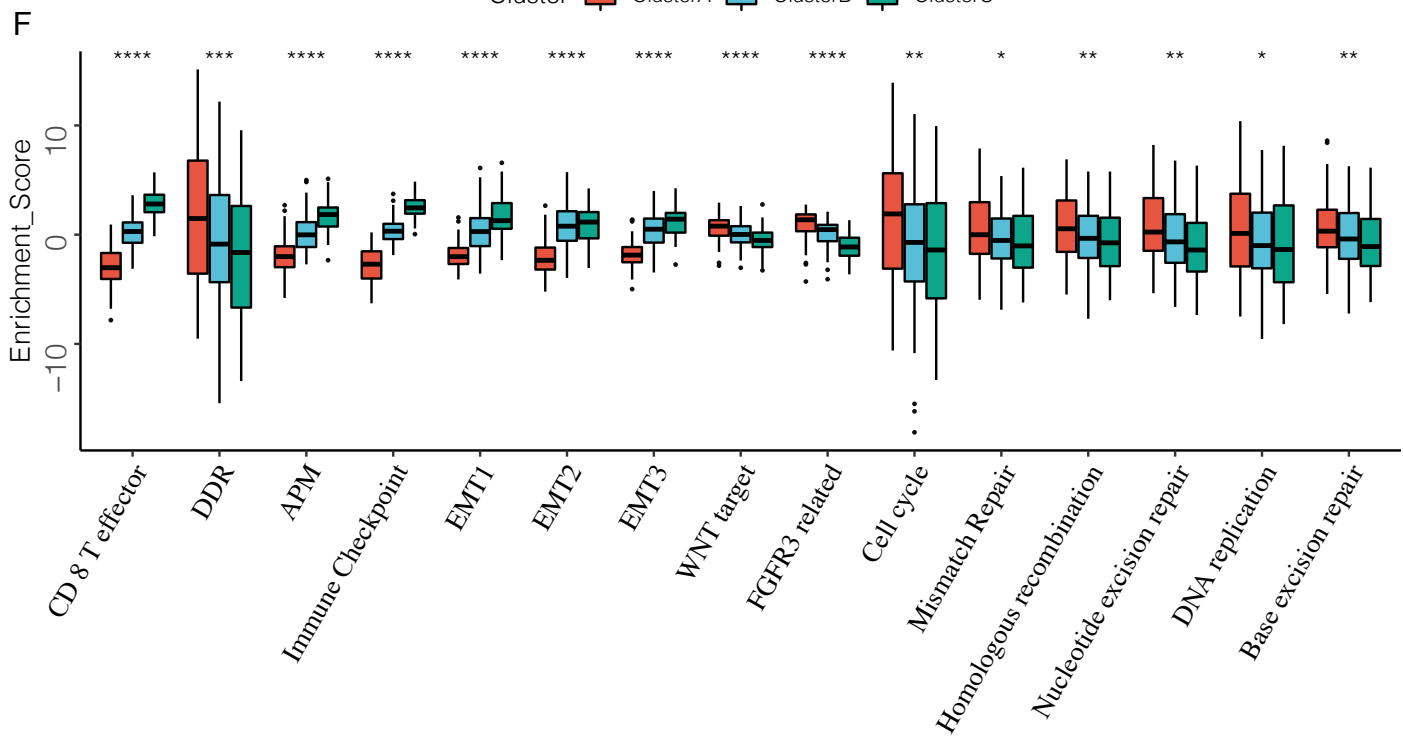

Supplement: Supplementary Figure 3 — Signal pathway differences and copy number characteristics among the three immunophenotypes. (A). The difference in Urothelial differentiation genes expression in the three immunotypes was compared using the Kruskal-Wallis test, and displayed by box plot. *P < 0.05; **P < 0.01; ***P < 0.001; ****P < 0.0001. (B). Using the Kruskal-Wallis test, we compared the differences in expression of related molecules of MHC I, MHC II, other MHC, co-inhibitors, and co-stimulators among three immunotypes. In the heat map, red represents upregulation and blue represents downregulation. *P < 0.05; **P < 0.01; ***P < 0.001; ****P < 0.0001. (C). The Kruskal-Wallis test was used to compare the score differences of 50 common biological gene sets in the three immunotypes and is displayed using a heat map. Red represents upregulation and blue represents downregulation. *P < 0.05; **P < 0.01; ***P < 0.001; ****P < 0.0001. (D). The figure shows the somatic mutations in the 20 genes with the highest frequency among the three immunophenotypes. Each column represents an individual patient. The upper panel shows tumor mutational burden, and the numbers on the right indicate the mutation frequency of each gene. We used different colors to represent different mutation types, and the small graph on the right shows the proportion of each mutation type. (E). Detailed cytobands with focal amplification (top) and focal deletion (bottom) in the three groups generated using the GISTIC2.0 software. The q value of each locus is plotted horizontally. [file Image_3.pdf]

**A**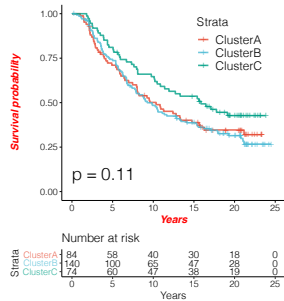**B**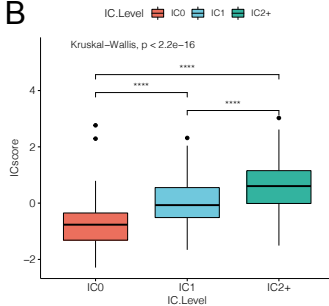**C**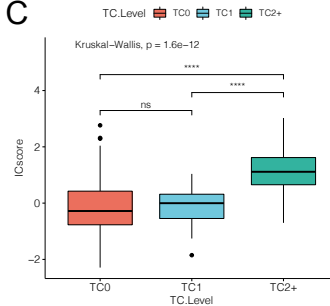**D**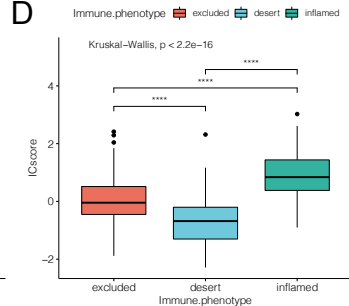**E**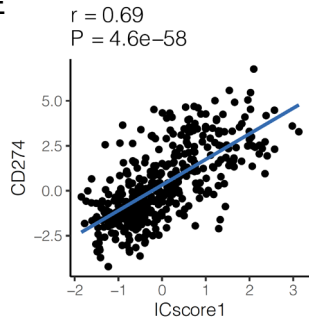**F**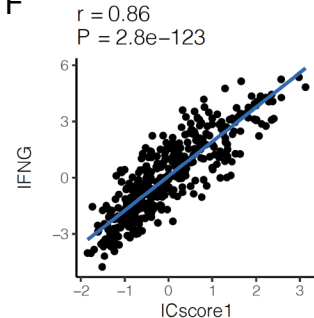**G**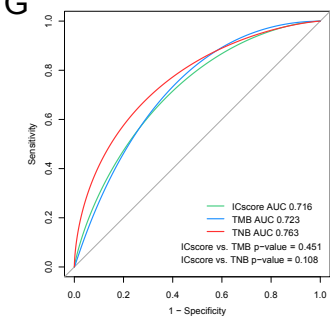**H**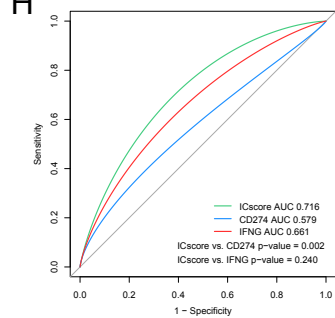

Supplement: Supplementary Figure 4 — The correlation among the three immunotypes and their response to immunotherapy. (A, B). The cluster stability was evaluated using the ConsensusClusterPlus R package, and the optimal number of clusters in the IMvigor210 cohort was determined to be 3. (C–F). The stacked histogram shows the three immunotypes and anti-programmed death-ligand 1 (PD-L1) efficacy (C), PD-L1 expression in immune cells (D), PD-L1 expression in tumor cells (E), and the defined immune subtype correlation (F). [file Image_4.pdf]

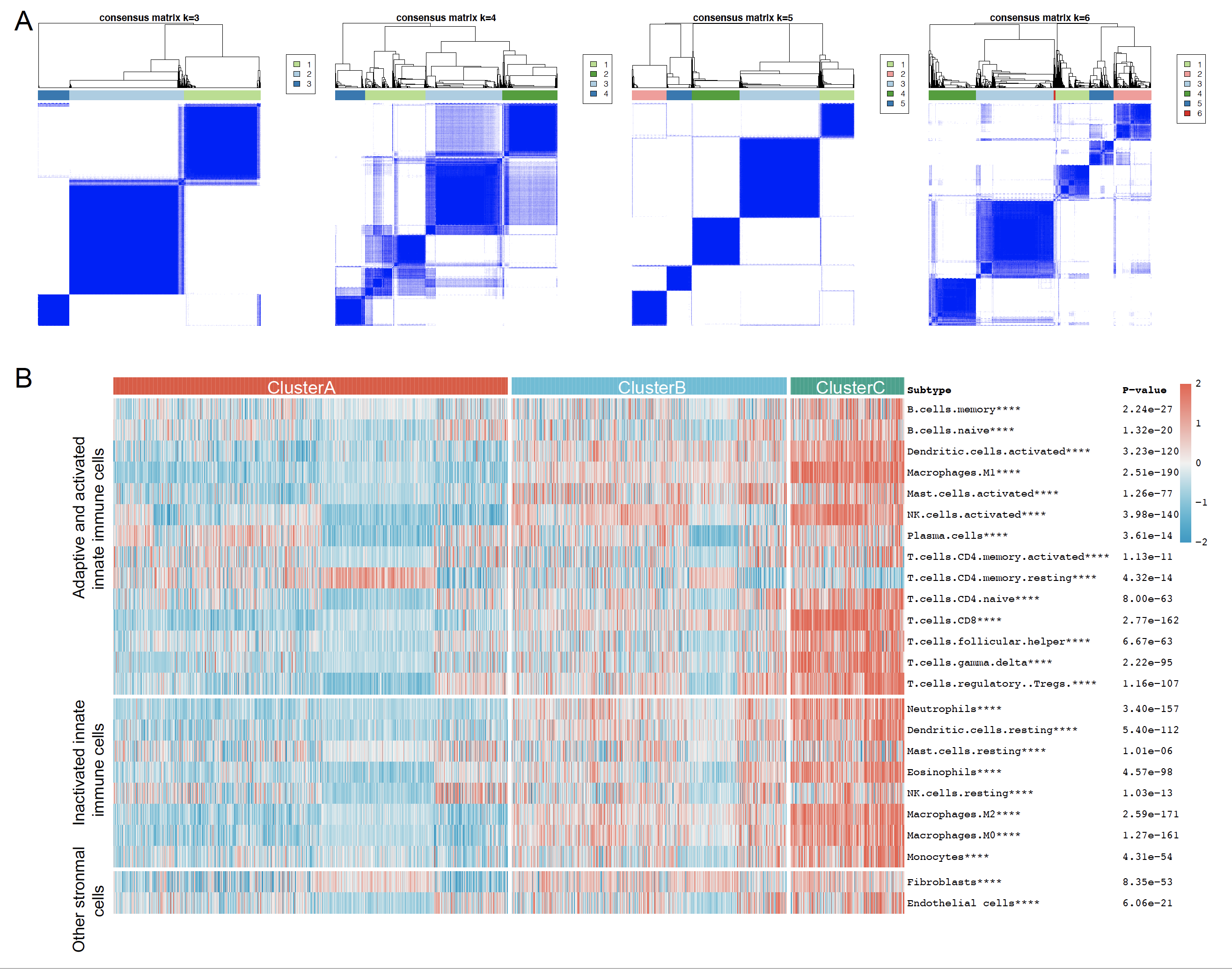

Supplement: Supplementary Figure 5 — ICscore is a reliable predictor of immune checkpoint inhibitor response. (A). Kaplan-Meier curve shows the prognostic significance of different immunotypes for overall survival in IMvigor210 cohort (OS; A) (B-D). The box plot shows the correlation between ICscore and programmed death-ligand 1 (PD-L1) expression in cells. (A), PD-L1 expression in cells (B), and the differences among the three defined immune subtypes. Kruskal-Wallis test was used to compare the differences between multiple groups, *P < 0.05; **P < 0.01; ***P < 0.001; ****P < 0.0001. (E, F). Spearman analyzed the correlation between ICscore, and CD274 and interferon γ in the IMvigor210 cohort. (G, H). Receiver operating characteristic (ROC) curve identified that ICscore can predict the responsiveness of anti-PD-L1 therapy well (area under the curve = 0.716). [file Image_5.png]
